# Sequence variation in ligand binding sites in proteins

**DOI:** 10.1186/1471-2105-6-240

**Published:** 2005-09-30

**Authors:** Thomas J Magliery, Lynne Regan

**Affiliations:** 1 Department of Molecular Biophysics & Biochemistry, Yale University, P.O. Box 208114, New Haven, CT 06520-8114, USA; 2 Present address: Department of Chemistry and Department of Biochemistry, The Ohio State University, 100 W. 18^th ^Ave., Columbus, OH 43210, USA; 3 Department of Chemistry, Yale University, New Haven, CT, USA

## Abstract

**Background:**

The recent explosion in the availability of complete genome sequences has led to the cataloging of tens of thousands of new proteins and putative proteins. Many of these proteins can be structurally or functionally categorized from sequence conservation alone. In contrast, little attention has been given to the meaning of poorly-conserved sites in families of proteins, which are typically assumed to be of little structural or functional importance.

**Results:**

Recently, using statistical free energy analysis of tetratricopeptide repeat (TPR) domains, we observed that positions in contact with peptide ligands are more variable than surface positions in general. Here we show that statistical analysis of TPRs, ankyrin repeats, Cys_2_His_2 _zinc fingers and PDZ domains accurately identifies specificity-determining positions by their sequence variation. Sequence variation is measured as deviation from a neutral reference state, and we present probabilistic and information theory formalisms that improve upon recently suggested methods such as statistical free energies and sequence entropies.

**Conclusion:**

Sequence variation has been used to identify functionally-important residues in four selected protein families. With TPRs and ankyrin repeats, protein families that bind highly diverse ligands, the effect is so pronounced that sequence "hypervariation" alone can be used to predict ligand binding sites.

## Background

The central challenge of the post-genomic era is to determine the structures and functions of thousands of newly-identified putative proteins [1,2]. Elucidating how proteins carry out their functions in diverse contexts and in organisms from all three domains of life is both fundamentally important to understanding biological function and critical for engineering new functions into novel proteins. Sequence conservation alone can be used to structurally categorize many proteins or putative proteins [3]. Additionally, catalytic sites in enzymes can sometimes be identified from conserved surface motifs [4-8]. In contrast, sites with poor sequence conservation have been largely ignored, because they are assumed to be of little structural or functional importance [9].

Sequence alignment of proteins with similar structures has shown that as sequence identity increases, average backbone variation decreases [10]. Within a particular protein family, residues in the hydrophobic core are the most conserved, implying they play a key role in specifying the fold [11]. In contrast, solvent-exposed residues tend to be variable, with mutations having little deleterious effect on overall structure or stability [12]. Consequently, conservation of surface residues is commonly taken to be an indication of functional importance [13]. This idea can be used to identify active-site residues from a collection of proteins that perform the *same *function, but it is not applicable to families of proteins that use a common scaffold to bind *diverse *ligands. Rather, we might hypothesize that such binding sites will be composed of positions that are variable.

Recently, we used a statistical free energy (SFE) approach [14,15] to understand better the role of conserved residues in defining tetratricopeptide repeat (TPR) motifs, which are thought to commonly mediate protein-protein interactions [16]. Strikingly, we found that the ligand-binding site of the motif can readily be identified by sequence hypervariation of positions proximal to the ligand, as evidenced by very low statistical free energies separating those positions from a position-independent reference state. Here, we examine this observation in more detail and demonstrate that specificity-determining residues in TPRs, ankyrin (Ank) repeats, Cys_2_His_2 _zinc fingers (Zifs), and PDZ domains can be identified from sequence variation.

By analyzing protein families with exceptional biochemical and biophysical characterization, we show that, when the ligand repertoire is highly diverse for a particular family, the binding site can be identified from sequence hypervariation alone. However, even when the ligands have significant features in common, sequence variation can be used to "dissect" binding sites to identify specificity-determining residues. We demonstrate this sequence variation using probabilistic and information theory approaches closely tied to the mathematics of covariation, which are more suitable for this type of analysis than SFEs or Shannon entropies. Statistical identification of specificity-determining residues will greatly facilitate the engineering proteins with novel functions and targets.

## Results & discussion

### The TPR binding site

**Figure 1:**
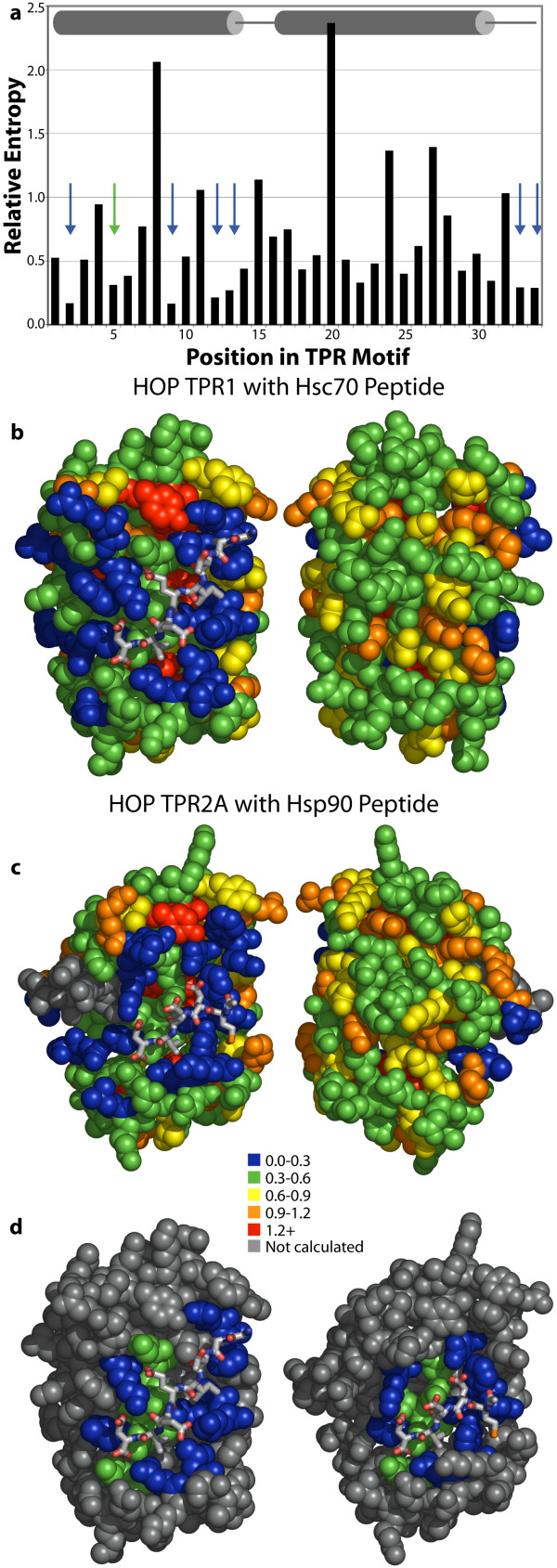
Relative entropy analysis of 6,887 canonical-length (34 aa) TPR repeats. (**a**) The relative entropy values are shown for each TPR position, with secondary structure indicated (cylinders represent helices and lines represent loops). Arrows indicate the positions of the seven most variable residues. These values are mapped onto the co-crystal structures of HOP-TPR1/Hsc70 peptide (**b**) and HOP-TPR2A/Hsp90 peptide (**c**), with the TPR domains rendered in spheres and the ligands in sticks. Two views from 180° rotation of each molecule are shown. The concave, ligand binding surfaces, left, are clearly more variable than the convex, solvent exposed surfaces, right. A small insertion in TPR2A is colored grey. (**d**) Views of the concave binding surfaces as in (**c**), but only those residues known to contact the ligand from co-crystal structures are colored [19]. Rendered from PDB entries 1ELW and 1ELR using PyMOL.

The TPR is a common 34 amino-acid protein motif that occurs in arrays, most frequently with three contiguous repeats [17]. Although TPR domains are thought to mediate protein-protein interactions, only a few examples have been well characterized. The large number of known TPR sequences, nearly 10,000 in Pfam [18], makes this motif an excellent target for statistical analysis. Using several mathematical approaches, we calculated the separation of the amino acid distribution at each position in TPRs from a position-independent reference state, amino acid usage in all proteins in yeast (Figure 1a). Note that we have performed this calculation on all of the 34 positions in the TPR motif; TPR domains are made up of tandem repeats of the TPR motif.

When relative entropy values are mapped onto the ligand-bound co-crystal structures of two different three-TPR domains (the TPR1 and TPR2A domains from Hsp-Organizing Protein, HOP [19]), it is immediately apparent that the concave, peptide-binding face of the TPR domains is *more variable *(*i.e.*, more like the reference state) than the convex, solvent-exposed face (Figure 1b and 1c). For clarity, the concave surfaces in Figure 1d are depicted with coloration of only ligand-binding residues independently identified from crystallographic analysis [19]. For both TPR domains, every residue in contact with the ligand peptide is in a position with a small relative entropy (blue or green in the figures), indicating small differences from the reference distribution.

This hypervariation is a consequence of each TPR having evolved to bind a *different *specific ligand (or portion of a ligand). When TPR proteins are considered collectively, the binding positions are statistically randomized to an extent that is dictated by the repertoire of amino acids required to perform the range of binding functions. In contrast, solvent-exposed residues in general mutate in a stochastic fashion throughout evolution, slowly reverting or "drifting" toward a "neutral" distribution. The lack of structural or functional importance of solvent-exposed residues results in little selective pressure against mutation, but the extent of randomization is limited by evolutionary time and subtle factors such as overall protein solubility. As a result of the high diversity of the ligands of TPR repeats, the binding surface is *more variable *than the solvent-exposed surface. Specifically, we predict that the ligand-binding residues that show the most sequence variation are the positions that determine ligand specificity.

Positions 2, 5, 9, 12, 13, 33 and 34 show the most sequence variation in TPRs (*i.e.*, they have the lowest relative entropies, ≤ 0.30). These seven residues all lie on the same face of the motif, and they are the residues that are exposed on the concave face of TPR domains. In fact, the TPR-peptide co-crystal structures show that residues in positions 2, 5, 6, 9, 12, and 13 are used by HOP TPR1 and TPR2A to bind their ligands [19]. Since few TPR-ligand structures have been solved, it remains to be seen whether or not other TPR domains utilize positions 33 and 34. Their spatial proximity to the other binding residues suggests that this is likely.

It is not surprising that some of the positions in contact with the ligand peptides are more biased than others (such as position 6). Some positions used for binding may also have other restrictions (such as structural restrictions) that limit the repertoire of amino acids allowed, and some positions may be important for binding affinity but not for specificity (which is to say they may bind a feature that is common among all ligands). For example, position 6 is modestly conserved in TPRs overall (it is frequently Asn) and is more buried than the other binding positions. The position-6 residues make contacts to the backbone of the ligand peptides here. It is worth noting that a position-6 Asn in the PEX5 C-terminal TPR domain also appears to make contact to the peptide backbone of the unrelated peroxisomal targeting signal peptide [20].

Several of the spheres in TPR2A are grey, indicating that they correspond to non-canonical positions and were therefore not calculated. At present, our analysis does not consider the effect of insertions and deletions. In the future, one could imagine including "deletion" as another "amino acid," so that site occupancy would contribute to the variation score.

### Measuring sequence variation

The use of metrics that measure the difference from a position-independent reference distribution is key to our observation, because (1) it is not clear how mere lack of conservation is related to variability, and (2) the likelihood of mutation from one residue to another is affected by factors such as genetic code bias and the greater difficulty of accommodating bulky or reactive amino acids. Here, we take the reference state to be amino acid usage in all open reading frames in *Saccharomyces cerevisiae *[21], which is independent of position but accounts for genetic code bias and amino acid chemistry. Using SFE calculations, we have previously demonstrated that using this reference state gives virtually indistinguishable results from other position-independent reference states such as amino acid usage in all proteins in the Pfam database [16].

We originally noted that the ligand binding site of TPRs was identified by sequence variation using statistical free energies [16]. SFEs are essentially a measure of the difference between amino acid distributions, relating the "probability" of observing a particular distribution to thermodynamic importance based on the exponential relationship given by the Boltzmann law.

PP(ref)=eΔGstatkT*     [1]
 MathType@MTEF@5@5@+=feaafeart1ev1aaatCvAUfKttLearuWrP9MDH5MBPbIqV92AaeXatLxBI9gBaebbnrfifHhDYfgasaacH8akY=wiFfYdH8Gipec8Eeeu0xXdbba9frFj0=OqFfea0dXdd9vqai=hGuQ8kuc9pgc9s8qqaq=dirpe0xb9q8qiLsFr0=vr0=vr0dc8meaabaqaciGacaGaaeqabaqabeGadaaakeaadaWcaaqaaiabdcfaqbqaaiabdcfaqnaaBaaaleaacqGGOaakcqWGYbGCcqWGLbqzcqWGMbGzcqGGPaqkaeqaaaaakiabg2da9iabdwgaLnaaCaaaleqabaWaaSaaaeaacqqHuoarcqWGhbWrdaWgaaadbaGaem4CamNaemiDaqNaemyyaeMaemiDaqhabeaaaSqaaiabdUgaRjabdsfaunaaCaaameqabaGaeiOkaOcaaaaaaaGccaWLjaGaaCzcaGqabiab=TfaBjab=fdaXiab=1faDbaa@4846@

(See Methods for an explanation of kT*.) This approach was introduced by Lockless & Ranganathan; however, their formalism for SFEs does not explicitly calculate the probability of observing a particular positional distribution [14]. Instead it uses the root-mean-square of the binomial probabilities of observing each amino acid, over all twenty amino acids *x*. That is,

ΔGstat=kT*∑x(ln⁡PxPx(ref))2     [2]
 MathType@MTEF@5@5@+=feaafeart1ev1aaatCvAUfKttLearuWrP9MDH5MBPbIqV92AaeXatLxBI9gBaebbnrfifHhDYfgasaacH8akY=wiFfYdH8Gipec8Eeeu0xXdbba9frFj0=OqFfea0dXdd9vqai=hGuQ8kuc9pgc9s8qqaq=dirpe0xb9q8qiLsFr0=vr0=vr0dc8meaabaqaciGacaGaaeqabaqabeGadaaakeaacqqHuoarcqWGhbWrdaWgaaWcbaGaem4CamNaemiDaqNaemyyaeMaemiDaqhabeaakiabg2da9iabdUgaRjabdsfaunaaCaaaleqabaGaeiOkaOcaaOWaaOaaaeaadaaeqbqaamaabmaabaGagiiBaWMaeiOBa42aaSaaaeaacqWGqbaudaWgaaWcbaGaemiEaGhabeaaaOqaaiabdcfaqnaaBaaaleaacqWG4baEcqGGOaakcqWGYbGCcqWGLbqzcqWGMbGzcqGGPaqkaeqaaaaaaOGaayjkaiaawMcaamaaCaaaleqabaGaeGOmaidaaaqaaiabdIha4bqab0GaeyyeIuoaaSqabaGccaWLjaGaaCzcaGqabiab=TfaBjab=jdaYiab=1faDbaa@5301@

where *P*_*x *_is given by

Px=N!nx!(N−nx)!fxnx(1−fx)N−nx     [3]
 MathType@MTEF@5@5@+=feaafeart1ev1aaatCvAUfKttLearuWrP9MDH5MBPbIqV92AaeXatLxBI9gBaebbnrfifHhDYfgasaacH8akY=wiFfYdH8Gipec8Eeeu0xXdbba9frFj0=OqFfea0dXdd9vqai=hGuQ8kuc9pgc9s8qqaq=dirpe0xb9q8qiLsFr0=vr0=vr0dc8meaabaqaciGacaGaaeqabaqabeGadaaakeaacqWGqbaudaWgaaWcbaGaemiEaGhabeaakiabg2da9maalaaabaGaemOta4KaeiyiaecabaGaemOBa42aaSbaaSqaaiabdIha4bqabaGccqGGHaqidaqadaqaaiabd6eaojabgkHiTiabd6gaUnaaBaaaleaacqWG4baEaeqaaaGccaGLOaGaayzkaaGaeiyiaecaaiabdAgaMnaaDaaaleaacqWG4baEaeaacqWGUbGBdaWgaaadbaGaemiEaGhabeaaaaGcdaqadaqaaiabigdaXiabgkHiTiabdAgaMnaaBaaaleaacqWG4baEaeqaaaGccaGLOaGaayzkaaWaaWbaaSqabeaacqWGobGtcqGHsislcqWGUbGBdaWgaaadbaGaemiEaGhabeaaaaGccaWLjaGaaCzcaGqabiab=TfaBjab=ndaZiab=1faDbaa@5462@

Here, *N *is the total number of sequences, *n*_*x *_is the number of sequences with amino acid *x *at the given position, and *f*_*x *_is the expected frequency of *x *from the reference state. This "vector" formalism for estimating the overall probability is empirically quite effective, but we speculated that a metric more tied to the mathematics of covariation would be more rigorous for our approach.

Since both of the following are true,

∑xnx=N     and     ∑xfx=1
 MathType@MTEF@5@5@+=feaafeart1ev1aaatCvAUfKttLearuWrP9MDH5MBPbIqV92AaeXatLxBI9gBaebbnrfifHhDYfgasaacH8akY=wiFfYdH8Gipec8Eeeu0xXdbba9frFj0=OqFfea0dXdd9vqai=hGuQ8kuc9pgc9s8qqaq=dirpe0xb9q8qiLsFr0=vr0=vr0dc8meaabaqaciGacaGaaeqabaqabeGadaaakeaadaaeqbqaaiabd6gaUnaaBaaaleaacqWG4baEaeqaaaqaaiabdIha4bqab0GaeyyeIuoakiabg2da9iabd6eaojaaykW7caaMc8UaaGPaVlaaykW7caaMc8UaeeyyaeMaeeOBa4MaeeizaqMaaGPaVlaaykW7caaMc8UaaGPaVlaaykW7cqqGGaaidaaeqbqaaiabdAgaMnaaBaaaleaacqWG4baEaeqaaaqaaiabdIha4bqab0GaeyyeIuoakiabg2da9iabigdaXaaa@5238@

the probability of observing a particular distribution is simply given by the multinomial probability,

Pmult=N!∏xnx!∏xfx     [4]
 MathType@MTEF@5@5@+=feaafeart1ev1aaatCvAUfKttLearuWrP9MDH5MBPbIqV92AaeXatLxBI9gBaebbnrfifHhDYfgasaacH8akY=wiFfYdH8Gipec8Eeeu0xXdbba9frFj0=OqFfea0dXdd9vqai=hGuQ8kuc9pgc9s8qqaq=dirpe0xb9q8qiLsFr0=vr0=vr0dc8meaabaqaciGacaGaaeqabaqabeGadaaakeaacqWGqbaudaWgaaWcbaGaemyBa0MaemyDauNaemiBaWMaemiDaqhabeaakiabg2da9maalaaabaGaemOta4KaeiyiaecabaWaaebuaeaacqWGUbGBdaWgaaWcbaGaemiEaGhabeaakiabcgcaHaWcbaGaemiEaGhabeqdcqGHpis1aaaakmaarafabaGaemOzay2aaSbaaSqaaiabdIha4bqabaGcdaahaaWcbeqaaiabd6gaUnaaBaaameaacqWG4baEaeqaaaaaaSqaaiabdIha4bqab0Gaey4dIunakiaaxMaacaWLjaWexLMBbXgBcf2CPn2qVrwzqf2zLnharyGvLjhzH5wyaGabbiaa=TfacaWF0aGaa8xxaaaa@563C@

**Figure 2:**
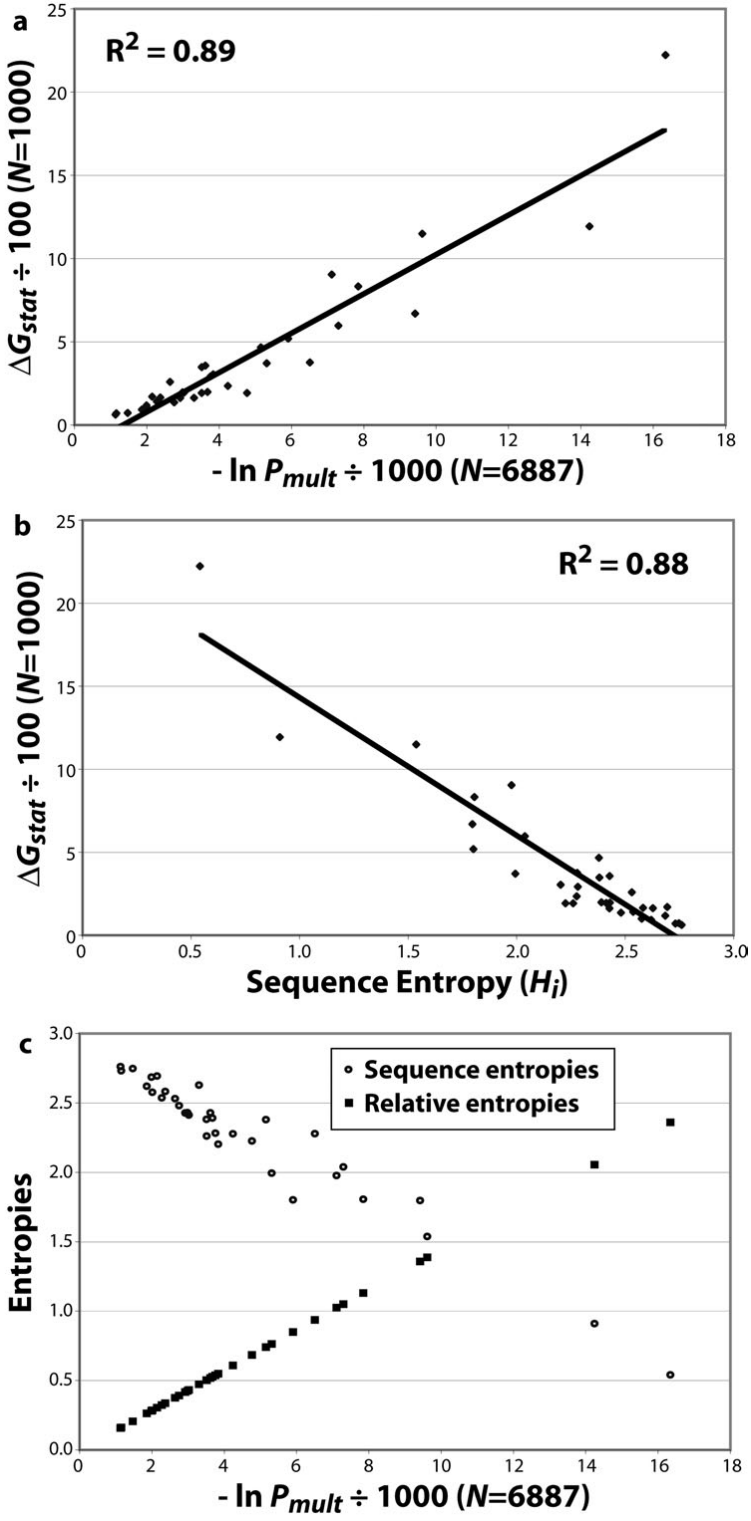
Measuring differences in distributions. (**a**) Lockless & Ranganathan statistical free energies versus the logarithm of the multinomial probability for each of the 34 sites in TPRs. (**b**) Relationship of SFEs to sequence (Shannon) entropy for TPR sites. (**c**) Relationship of logarithm of multinomial probabilities to sequence entropy (circles) and relative entropy (squares).

As expected, the Δ*G*_*stat *_values are very closely related (R^2 ^= 0.89) to the ln *P*_*mult *_values for the 34 positions in the TPR motif (Figure 2a). Note, however, that the values of Δ*G*_*stat *_and ln *P*_*mult *_are dependent upon the total number of sequences *N *(since it is much less likely to observe a particular amino acid 200 times out of 1000 than 20 out of 100, if you are expecting it only 5% of the time).

Recently, Dekker et al. suggested that SFEs are merely a measure of sequence (Shannon) entropy (*H*_*i*_), which implicitly measures how a distribution varies from equal usage [22]. (This is because the maximum entropy arises from a distribution with equal usage.)

Hi=−∑xpxln⁡px     [5]
 MathType@MTEF@5@5@+=feaafeart1ev1aaatCvAUfKttLearuWrP9MDH5MBPbIqV92AaeXatLxBI9gBaebbnrfifHhDYfgasaacH8akY=wiFfYdH8Gipec8Eeeu0xXdbba9frFj0=OqFfea0dXdd9vqai=hGuQ8kuc9pgc9s8qqaq=dirpe0xb9q8qiLsFr0=vr0=vr0dc8meaabaqaciGacaGaaeqabaqabeGadaaakeaacqWGibasdaWgaaWcbaGaemyAaKgabeaakiabg2da9iabgkHiTmaaqafabaGaemiCaa3aaSbaaSqaaiabdIha4bqabaGccyGGSbaBcqGGUbGBcqWGWbaCdaWgaaWcbaGaemiEaGhabeaaaeaacqWG4baEaeqaniabggHiLdGccaWLjaGaaCzcamXvP5wqSXMqHnxAJn0BKvguHDwzZbqegyvzYrwyUfgaiqqacaWFBbGaa8xnaiaa=1faaaa@4C11@

Here, *p*_*x *_is the proportion of sequences with amino acid *x *at position *i*. While it is true that Δ*G*_*stat *_is correlated with *H*_*i *_(Figure 2b), this correlation is a consequence of the reference state of Δ*G*_*stat *_calculations being fairly close to equal usage of amino acids. It is also affected by the fact that Δ*G*_*stat *_values are not based on a rigorous measure of overall probability.

Plotting the multinomial probabilities associated with the distributions at the 34 positions in TPRs against sequence entropy and relative entropy clearly demonstrates that ln *P*_*mult *_measures *the same thing *as relative entropy, but something different from sequence entropy (Figure 2c). (The difference becomes more dramatic the more the reference state deviates from equal usage.) In fact, relative entropy *D*(*p*||*f*) is an information theory approach to measuring the "distance" between distributions, given by,

D(p||f)=∑xpxln⁡pxfx     [6]
 MathType@MTEF@5@5@+=feaafeart1ev1aaatCvAUfKttLearuWrP9MDH5MBPbIqV92AaeXatLxBI9gBaebbnrfifHhDYfgasaacH8akY=wiFfYdH8Gipec8Eeeu0xXdbba9frFj0=OqFfea0dXdd9vqai=hGuQ8kuc9pgc9s8qqaq=dirpe0xb9q8qiLsFr0=vr0=vr0dc8meaabaqaciGacaGaaeqabaqabeGadaaakeaacqWGebarcqGGOaakcqWGWbaCcqGG8baFcqGG8baFcqWGMbGzcqGGPaqkcqGH9aqpdaaeqbqaaiabdchaWnaaBaaaleaacqWG4baEaeqaaOGagiiBaWMaeiOBa42aaSaaaeaacqWGWbaCdaWgaaWcbaGaemiEaGhabeaaaOqaaiabdAgaMnaaBaaaleaacqWG4baEaeqaaaaaaeaacqWG4baEaeqaniabggHiLdGccaWLjaGaaCzcamXvP5wqSXMqHnxAJn0BKvguHDwzZbqegyvzYrwyUfgaiqqacaWFBbGaa8Nnaiaa=1faaaa@5410@

It can be shown, using the Stirling approximation for factorials, that multinomial probability is in fact linearly related to relative entropy by the number of sequences (see Supplemental Material).

One significant advantage of relative entropy over multinomial probability and Δ*G*_*stat *_is that relative entropy is *independent *of the total number of sequences *N*. Since the intention of the SFE approach is to estimate the significance associated with an amino acid distribution relative to a reference state, we submit that relative entropies are the most convenient way to do this. Relative entropies combine the sample-size independence and ease of calculation of Shannon entropies with the reference-state correction of Lockess & Ranganathan's method, while at the same time measuring that correction in a mathematically-rigorous way.

Other methods, in addition to Shannon entropy and the Lockless & Ranaganthan method, have been suggested for scoring residue conservation, including metrics that account for residue properties such as size or hydrophobicity [23]. There is evidence that binding sites have unique compositional preferences, which may suggest alternative reference states for our method [24]. It will be interesting to examine how attention to property variation may improve our method of dissecting binding sites in structural families.

### Effects of sample size

**Figure 3:**
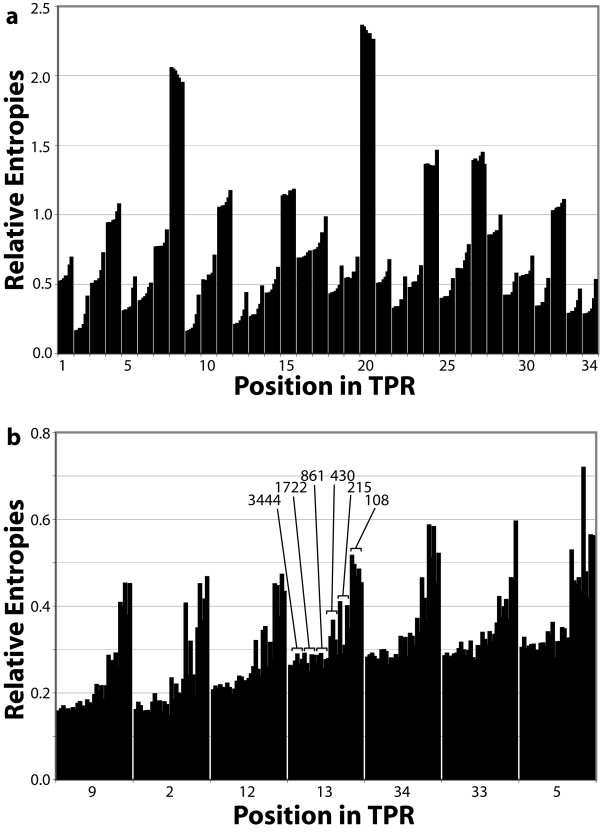
Effects of sample size. (**a**) Average relative entropy associated with each of the 34 positions in TPRs with random subsets of various sizes. Each cluster of bars represents one position in the TPR motif. The cluster is composed of bars, left to right, from sets with approximately 6887, 3444, 1722, 861, 430, 215 and 108 sequences. Each bar is the average of five subsets of the same size (except 6887, since there is only one set this size – all sequences). (**b**) Relative entropies associated with five randomly chosen subsets of various sizes for the seven positions most like the reference state. Each cluster of bars represents one position. The individual bars show the calculated relative entropies for subsets of the same sizes as in (**a**) (five of each size).

In order to determine how many sequences are required to identify binding residues in TPRs by this method, subsets of various sizes were randomly selected from the 6,887 TPR sequences. In Figure 3a, the average relative entropy values from subsets (5 each) with approximately 6887, 3444, 1722, 861, 430, 215 and 108 randomly-selected sequences are shown for all positions. The overall pattern is evident with as few as about 100 sequences, and there is virtually no difference between subsets with 400 or more sequences. Values from the five random subsets of each size are shown for the seven lowest relative entropy positions in the full data set (Figure 3b). Again, there is essentially no discernable difference down to as few as 400 sequences, and there is not significant variability until one examines fewer than 200 sequences. We therefore expect that this analysis is applicable to protein families with as few as about 200–400 sequences.

### Ankyrin repeats: comparison with experiment

**Figure 4:**
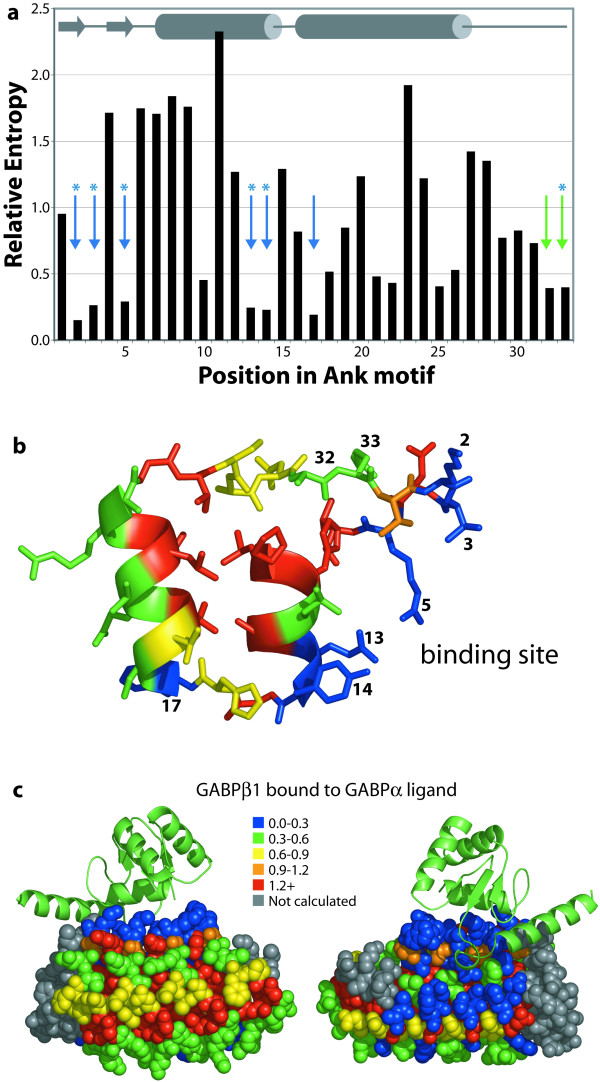
Relative entropy analysis of canonical positions in 15,497 Ank repeats. (**a**) The positional relative entropies are shown with secondary structural elements noted (grey arrows are β-strands). Blue and green arrows indicate the most variable positions; asterisks (*) indicate positions mutated by the Plückthun lab to alter Ank-domain specificity. (**b**) The location of the binding site in a single Ank repeat in the loop and proximal α-helical surface is labeled. (**c**) The 4-Ank domain from GABPβ1 (spheres) is shown bound to the ligand GABPα (ribbons) in two views from 180° rotation. Again, the binding surface is evident from the low relative entropies. Note that some non-binding surface-exposed positions, particularly turn residues, are conserved due to their importance in defining the Ank fold. Some positions in GABPβ1 do not map onto the canonical Ank sequence and are colored grey. Rendered using PyMOL from PDB entry 1AWC.

A corresponding analysis of ankyrin (Ank) repeats, another experimentally well-characterized protein-binding motif, clearly confirms our prediction that low relative entropies can be used to identify specificity-determining residues when the repertoire of ligand is highly variable. Anks are helix-turn-helix-loop motifs, which bind their ligands with residues in the loops and on the surface of the helical array proximal to the loops [25]. Figure 4 shows the relative entropies from over 15,000 Ank repeats mapped onto the co-crystal structure of mouse GA binding protein β1 with the GABPα ligand bound [26]. Again, our analysis dramatically reveals that the residues known to form the binding site are among the most variable; the positions most like the reference state (relative entropy ≤ 0.39) are 2, 3, 5, 13, 14, 17, 32, and 33.

Significantly, the functional importance of the residues we have identified has already been verified experimentally. Plückthun and colleagues considered four Ank-ligand co-crystal structures and chose key interacting residues in Ank repeats from those whose solvent accessibility was most changed upon ligand binding: positions 2, 3, 5, 13, 14, and 33. Ank repeat domains which bind to different ligands, for example maltose binding protein and two kinases, have been selected from a library of Ank repeat domains in which only these positions were randomized [27,28]. This result confirms our proposal that these are the specificity-determining residues. In fact, the crystal structure of one of the selected ankyrin proteins that binds MBP directly demonstrates the role of these residues in binding. In addition, position 32 is close in space to these residues, and may well participate in binding for some Ank proteins. It is not clear why position 17, which lies in the turn between the motif's helices, is more variable than other non-binding surface-exposed positions.

### Dissecting binding sites when ligands have conserved features

**Figure 5:**
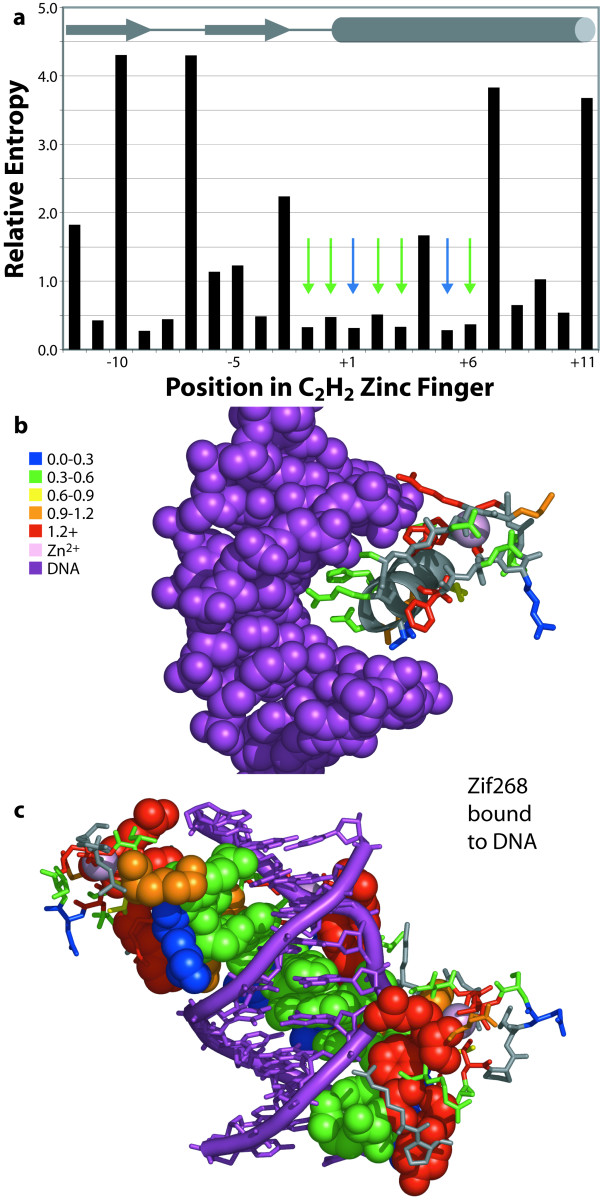
Relative entropy analysis of canonical positions in 28,442 C_2_H_2 _zinc fingers. (**a**) Positions in the graph are shown in the order found in Pfam and numbered by convention (where -1 is the residue N-terminal to the α-helix). Note that the y-axis scale is different from Figs. 1 and 2 due to the almost invariant zinc(II)-binding residues (-10, -7, +7 and +11). Blue and green arrows indicate the seven predicted specificity-determining positions. (**b**) The middle zinc finger of Zif268 bound to DNA (purple) is shown, with the Zn(II) atom as a pink sphere [43]. (**c**) The residues in contact with the DNA from all three zinc fingers of Zif268 are rendered in spheres. The DNA-binding positions group into variable, specificity-determining positions (blue and green spheres) projecting into the major groove of the DNA, and conserved positions that enhance affinity to DNA but do not affect specificity (orange and red spheres). Rendered with PyMOL from PDB entry 1A1I.

We also analyzed Cys_2_His_2 _zinc fingers (Zifs) in the same way. In contrast to TPR and Ank repeats, which are stabilized by burial of hydrophobic residues, Zifs are mostly stabilized by ligation of zinc(II) and binding to DNA. Not surprisingly, then, positions that ligate the zinc ion (Cys_-10_, Cys_-7_, His_+7_, His_+11_) and a subset of positions that contact the DNA (*e.g.*, Tyr_-12_, Lys_-5_, Phe_-3_, Arg_+9_) are highly biased (where -1 is the position immediately before the α-helix and the consensus residue is listed). Residues with low relative entropy (≤0.5) are essentially in two patches, near the end of the end of the helix buried in the major groove and on the solvent-exposed surface distal to the DNA. When one considers only the positions that are in contact with the ligand DNA, the residues with the lowest relative entropies (blue and green spheres in Figure 5) are positions -2, -1, 1, 2, 3, 5 and 6. Extensive phage-display selection work has shown that positions -1, 1, 2, 3, 5 and 6 are critical to specificity for the target DNA sequences studied [29,30]. In contrast, positions that contact the DNA but have no effect on specificity, such as basic residues that make contacts to the phosphate backbone, are essentially invariant (orange and red spheres in Figure 5).

Note that in this case the specificity-determining residues are not necessarily the most variable residues in the motif; they are the most variable residues in the motif *that are in contact with the ligand*. Thus, for Zifs, sequence hypervariation is not sufficient to identify the binding site, but statistical analysis together with a sample structure reveals specificity-determining positions without further characterization. Apparently, the repertoire of amino acids needed to bind DNA in the major groove is less diverse than that needed for the range of binding functions exhibited by TPR or Ank repeat domains.

**Figure 6:**
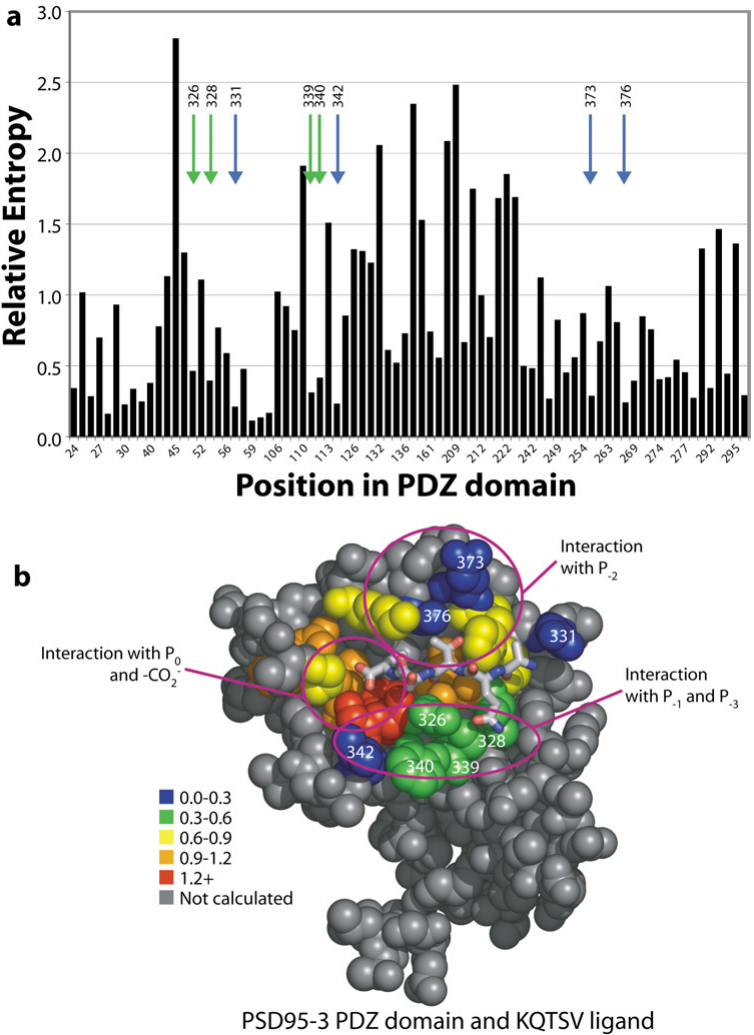
Relative entropy analysis of canonical positions in 2,751 PDZ domains. **(a) **Positions in the graph are shown in the order found in Pfam and with the same numbering. Only positions with greater than 50% occupancy were calculated. The eight variable binding positions are marked with arrows, and the corresponding residue number in PSD95-3 is listed. **(b) **The structure of PSD95-3 with its cognate ligand peptide, KQTSV [44]. Note that atoms are missing from the ligand lysine sidechain due to lack of electron density in the X-ray data. The structurally-determined binding residues (see text) are colored, and the eight predicted specificity-determining positions are labeled with residue numbers as in (a). Rendered with PyMOL from PDB entry 1BE9.

A similar phenomenon is observed for PDZ domains, whose peptide ligands have highly conserved elements. PDZ domains are ubiquitous globular protein-protein interaction domains. It is thought that most PDZ domains bind to the C-terminus of target proteins, typically making contact to the carboxyl-terminal four to five residues. PDZ domains can be categorized into two classes (I and II) that bind to consensus sequences X-(S/T)-X-(V/I/L)-CO_2_^- ^and X-Φ-X-Φ-CO_2_^-^, respectively (where Φ represents a hydrophobic residue and X represents an arbitrary residue) [31]. Since our method identifies residues that vary with the repertoire of ligands (i.e., specificity-determining residues), we would expect that positions that bind to the terminal carboxylate and C-terminal hydrophobic side-chain (P_0_) will be highly biased (i.e., conserved); positions that bind to the alcohol or hydrophobic residue at P_-2 _will be biased; and positions that bind to P_-1 _and P_-3 _will be highly variable. In Figure 6b, we have highlighted the positions in the example PDZ domain 3 from PSD95 that have been identified from NMR studies and X-ray co-crystal structures to be involved in binding to the terminal four residues of the ligand (323–328, 339, 340, 342, 372, 373, 376, 379 and 380) [32]. We also included positions 318, 322, 329 and 331, which are within 5 Å of the ligand peptide (KQTSV-CO_2_^-^) in the example structure (computed with DeepView [33]).

The eight most variable residues in this group are 331, 342, 376, 373, 339, 328, 340 and 326. The most variable residue, 331 (here, a glutamate), contacts the P_-4 _lysine, which is variable among PDZ ligands and whose effect on specificity has not been extensively examined. Positions 326, 328, 339, 340 and 342 interact with the variable P_-1 _and P_-3 _positions. Interestingly, computational redesign of PDZ domain specificity confirms the central importance of these residues in specificity determination. Reina et al. were able to change the specificity of PSD95-3 from KQTSV to KITWV and KRTEV (retaining ligand class, since P_0 _and P_-2 _are the same) [34]. In the first case, residues 326, 339, 340, 342, 380 and 397 were mutated. In the second case, residues 326, 328, 339, 340, 342 and 397 were mutated. Note that position 397 is outside of the canonical PDZ domain, and so was not examined in our work; position 380 was mutated to improve the stability of the domain, not its binding specificity.

Positions 373 and 376 contact the P_-2 _position, which is either an alcohol or a hydrophobic residue depending upon class. The identity of position 372 is known to be highly correlated with the ligand class, because it is typically occupied by a polar residue (often histidine) for class I ligands and by a hydrophobic residue for class II ligands [31]. As expected, position 372 displays intermediate variability (yellow in the figure). We hypothesize that positions 373 and 376 are much more variable than 372 and 380 because they are further away from the P_-2 _threonine; in fact, position 373 is farther than 5 Å away. These residues are likely more important for binding class II ligands in which P_-2 _is hydrophobic and therefore generally larger. P_0 _is a hydrophobic residue in virtually every known PDZ ligand, and it invariantly presents the carboxylate terminus of the peptide. Not surprisingly, then, the residues that it contacts (322–325, 327) are highly biased. In fact, position 324 is a glycine in 97% of PDZ domains, and the turn in which it lies hosts the carboxylate.

It is worth noting that, in contrast to the other three examples presented above, this calculation was carried out on a whole domain instead of a repeat motif. It is also worth noting that the binding site, as in the case of zinc fingers, could not be easily predicted from relative variability of sequence alone due to commonalities among the ligands that result in conserved elements of the binding sites. However, in combination with an example co-crystal structure, the specificity-determining positions can again be inferred from sequence variation, and the inference matches closely what has been derived from extensive biochemical characterization and engineering.

### The meaning of relative entropy values

In our previous study of TPRs by SFE analysis, we empirically demonstrated that for a particular sample size and scaling, levels of sequence variation (Δ*G*_*stat*_) could be usefully grouped as such: 0–1.25, hypervariable or no bias; 1.25–2.5, slight bias; 2.5–5.0, significant bias; 5.0–10.0, dramatic bias; 10+, restriction to a small subset of amino acids [16]. Regression analysis between SFE and relative entropy values for all TPR positions suggests that these values correspond to relative entropies of approximately 0.3, 0.5, 0.9 and 1.5. For convenience, we therefore chose 0.0–0.3, 0.3–0.6, 0.6–0.9, 0.9–1.2 and 1.2+ as bins for coloration of the figures in this publication. The examples in this study suggest that "normal" surface positions typically exhibit relative entropies in the range of 0.3–0.6, and that specificity-determining positions typically have relative entropies less than 0.5. The overlap of these values highlights the difficulty in using this approach as a purely predictive algorithm: only when the repertoire of ligands is extremely diverse (as with TPRs and ankyrin repeats) is there a clear distinction between ligand-binding residues and surface residues in general. We are in the process of a much broader application of this procedure to all of the families in the Pfam database, which we will use to refine the meaning of the relative entropy values in protein families overall (M. Gerstein, T. Gianoulis, T.J.M. and L.R., unpublished work).

## Conclusion

The notion that positions that bind diverse ligands will be variable among a family of proteins seems fairly obvious, but this approach has not yet been utilized as a general strategy. One notable precedent is seen in the original studies of antigen-binding sites in antibodies, which were identified as variable regions when the amino acid sequences of antibodies were first determined [35]. Various family-based approaches have been applied to the prediction of functional residues, typically analyzing sequence variability from collections of proteins with similar function (and therefore emphasizing the functional importance of *conserved *residues). For example, "evolutionary trace" and related methods divide multiply-aligned sequences into subfamilies, typically by phylogeny, comparing patterns of conservation among evolutionarily-related subfamilies and often mapping onto 3D structure [13,36-42]. Basically, these methods posit that positions that are conserved in all sequences are important for structure, and positions that are conserved within subfamilies (but vary among the sub-types) are important for function (i.e., the function of the proteins in the subfamily).

Here we show how analysis of sequence variability can be enlarged to understand functional variability in *whole families *of proteins with similar structures. If one collects proteins with the same structure and diverse functions, then structural positions will be conserved and functional positions will vary, and the degree of variation will be related to the degree of variation among the ligands or substrates. In the case of repeat motifs such as TPRs and ankyrins, where structural elements are further divorced from functional conservation by ignoring how the motifs are arranged in domains, the degree of variability is sometimes so profound that it alone can be used to predict the binding site. When the ligands have commonalities, then it becomes more difficult to predict the binding site from variation alone. In that case, as with PDZ domains and Zifs, variation in combination with an example structure still reveals specificity-determining binding positions, which is critical information for re-engineering specificity. (The corollary to this argument is that the pattern of variation of known binding residues will suggest the pattern of variation in the ligands.)

Analysis of overall variance among structurally-related families provides complementary information to methods that analyze variance among evolutionarily-related subfamilies, which have proven very powerful in recent years. A major challenge for these evolutionary trace methods is accurate functional sub-typing, particularly when family members have diverged very significantly. Our method avoids functional sub-typing and, rather, benefits from increased functional divergence of family members (since it results in increased variation among functional positions). Further attention to variable residues in families overall therefore stands to improve exiting methods of functional prediction.

There are hundreds of binding scaffolds with sufficient examples known to permit this type of statistical analysis. The use of rigorous measures of how amino acid distributions differ improves significantly upon conservation alone as a means of identifying important residues within a protein family (this has been reviewed recently [23]). The rapid identification of specificity-determining positions will be useful for the design of proteins with altered binding specificity. The predictions of specificity-determining residues in Ank repeat proteins, Zifs and PDZ domains agree strikingly well with results from considerable structural and biochemical work, and therefore provide a guide for re-engineering binding specificity by design even for protein families lacking extensive characterization. Moreover, knowledge of the specificity-determining residues can be incorporated into evolutionary trace methods to develop a comprehensive view of residues critical for function.

## Methods

### Sequences

Aligned sequences of TPRs, Ank repeats, C_2_H_2 _Zifs and PDZ domains were downloaded from Pfam [18]. TPRs of non-canonical length (*i.e.*, not 34 amino acids) were discarded, and only canonical positions were considered with Ank repeats, Zifs and PDZ domains (*i.e.*, ignoring low-occupancy positions from insertions and deletions). All calculations were carried out in Microsoft Excel 2003 on a Dell Latitude C640 with a 2.2 GHz Intel Mobile Pentium 4 processor. Factorials were computed from the Stirling approximation.

### Statistical Free Energies

The SFEs associated with each amino acid were determined from application of the Boltzmann law [1], where *k *is the Boltzmann constant, kT* is an arbitrary energy unit (since the "temperature" of the ensemble T* is not necessarily related to *T *for conventional systems), and *P*_*ref *_is the probability associated with a hypothetical site with amino acid usage as in the reference state. The binomial probability *P*_*x *_of the observation of *n*_*x *_sequences with amino acid *x *was calculated from [3], where *f*_*x *_is the frequency of the amino acid in a reference state, all ORFs in *Saccharomyces cerevisiae*. The SFEs associated with the observed frequencies of the 20 amino acids at each site can be thought of as elements of a 20-dimensional vector. The scalar length of this vector, the root-mean-square average for all amino acids [2], is therefore taken to be the statistical free energy, Δ*G*_*stat*_, that separates the observed positional amino acid distribution from the reference state. For comparison to Lockless & Ranganathan [14], the Δ*G*_*stat *_values were arbitrarily divided by 100. However, the Ranganathan group normalizes the number of sequences to 100, and we have shown that Δ*G*_*stat *_is proportional to *N *for large *N*. Therefore, the Δ*G*_*stat *_values we calculate are 10-fold larger than those calculated by the Ranganathan method for the same amino acid distribution, since we have normalized to *N *= 1000.

### Other metrics of distribution difference

Multinomial probabilities [4], sequence (Shannon) entropies [5] and relative entropies [6] were calculated as described above. For sequence and relative entropy calculations, the frequencies were calculated as:

px=nx+1N+1     [7]
 MathType@MTEF@5@5@+=feaafeart1ev1aaatCvAUfKttLearuWrP9MDH5MBPbIqV92AaeXatLxBI9gBaebbnrfifHhDYfgasaacH8akY=wiFfYdH8Gipec8Eeeu0xXdbba9frFj0=OqFfea0dXdd9vqai=hGuQ8kuc9pgc9s8qqaq=dirpe0xb9q8qiLsFr0=vr0=vr0dc8meaabaqaciGacaGaaeqabaqabeGadaaakeaacqWGWbaCdaWgaaWcbaGaemiEaGhabeaakiabg2da9maalaaabaGaemOBa42aaSbaaSqaaiabdIha4bqabaGccqGHRaWkcqaIXaqmaeaacqWGobGtcqGHRaWkcqaIXaqmaaGaaCzcaiaaxMaatCvAUfeBSjuyZL2yd9gzLbvyNv2CaeHbwvMCKfMBHbaceeGaa83waiaa=DdacaWFDbaaaa@46FA@

so that ln *p*_*x *_was always defined (and is valid as long as *N *is large). The values used for *f*_*x *_are listed in our previous paper [16].

## List of abbreviations

Ank, ankyrin; HOP, Hsp-organizing protein; MBP, maltose binding protein; SFE, statistical free energy; TPR, tetratricopeptide repeat protein; Zif, zinc finger

## Authors' contributions

T.J.M. performed all calculations and analysis, and drafted the manuscript. L.R. aided in interpretation of the data and manuscript preparation, and provided support.

## Supplementary Material

Additional File 1 The relationship between multinomial probability and relative entropy is derived. Click here for file
