# Supplementary material for: Sequence variation in ligand binding sites in proteins
Source: BMC Bioinformatics. 2005 Sep 30;6:240. doi: 10.1186/1471-2105-6-240 (PMC1261162; doi:10.1186/1471-2105-6-240)
Supplement: Additional File 1 — The relationship between multinomial probability and relative entropy is derived. [file 1471-2105-6-240-S1.pdf]

Sequence variation in ligand binding sites in proteins

Thomas J. Magliery[1,2*] and Lynne Regan[1,3*]
Departments of Molecular Biophysics & Biochemistry[1] and Chemistry[3], New Haven, Connecticut 06520, U.S.A.
[2]Present Address: Departments of Chemistry and Biochemistry, The Ohio State University, 100 W. 18th Ave., Columbus, Ohio 43210, U.S.A.
[*]To whom correspondence should be addressed. TJM: E-mail magliery@chemistry.ohio-state.edu. Phone +1 (614) 247-8425. Fax +1 (614) 292-1685. LR: E-mail lynne.regan@yale.edu. Phone +1 (203) 432-9843. Fax +1 (203) 432-5767.

Supplementary Material

The relationship between multinomial probability and relative entropy can be demonstrated using the Stirling approximation for factorials:

$$\ln x! \approx (x + \frac{1}{2})\ln x - x + \frac{1}{2}\ln 2\pi \approx x \ln x - x$$

Taking the natural log of the multinomial probability gives:

$$\ln P_{mult} = \ln N! - \ln n_1! - \ln n_2! ... + n_1 \ln f_1 + n_2 \ln f_2 ...$$

Substitution of the factorials with the Stirling approximation gives:

$$\ln P_{mult} = N \ln N - N - (n_1 \ln n_1 - n_1) ... + n_1 \ln f_1 ...$$

Since the sum of n_x over all x is N, these cancel to give:

$$\ln P_{mult} = N \ln N - \sum_x n_x \ln \frac{n_x}{f_x}$$

Subtracting ($n_x \ln N$) from each term in the summation, which sums to ($N \ln N$) over all x, gives:

$$\ln P_{mult} = -\sum_x n_x \ln \frac{p_x}{f_x}$$

Where $p_x = n_x/N$. Therefore,

$$\ln P_{mult} = -N \sum_x p_x \ln \frac{p_x}{f_x} = -N \times D(p \,||\, f)$$

In other words, dividing the natural log of the multinomial probability by the total number of sequences gives the negative of the relative entropy.
